# Finerenone attenuates downregulation of the kidney GLP-1 receptor and glucagon receptor and cardiac GIP receptor in mice with comorbid diabetes

**DOI:** 10.1186/s13098-024-01525-3

**Published:** 2024-11-24

**Authors:** Duc Tin Tran, Emily S. H. Yeung, Lisa Y. Q. Hong, Harmandeep Kaur, Suzanne L. Advani, Youan Liu, Madiha Zahra Syeda, Sri Nagarjun Batchu, Andrew Advani

**Affiliations:** Keenan Research Centre for Biomedical Science, St. Michael’s Hospital, 6-151 61 Queen Street East, Toronto, ON M5C 2T2 Canada

**Keywords:** CCN2, CTGF, Diabetic nephropathy, Finerenone, Gastric inhibitory polypeptide, GLP-1, GLP-1 receptor, Glucagon, Glucagon receptor, Mineralocorticoid receptor

## Abstract

**Background:**

Several new treatments have recently been shown to have heart and kidney protective benefits in people with diabetes. Because these treatments were developed in parallel, it is unclear how the different molecular pathways affected by the therapies may overlap. Here, we examined the effects of the mineralocorticoid receptor antagonist finerenone in mice with comorbid diabetes, focusing on the regulation of expression of the glucagon-like peptide-1 receptor (GLP-1R), gastric inhibitory polypeptide receptor (GIPR) and glucagon receptor (GCGR), which are targets of approved or investigational therapies in diabetes.

**Methods:**

Male C57BL/6J mice were fed a high fat diet for 26 weeks. Twelve weeks into the high fat diet feeding period, mice received an intraperitoneal injection of streptozotocin before being followed for the remaining 14 weeks (DMHFD mice). After 26 weeks, mice were fed a high fat diet containing finerenone (100 mg/kg diet) or high fat diet alone for a further 2 weeks. Cell culture experiments were performed in primary vascular smooth muscle cells (VSMCs), NRK-49 F fibroblasts, HK-2 cells, and MDCK cells.

**Results:**

DMHFD mice developed albuminuria, glomerular mesangial expansion, and diastolic dysfunction (decreased E/A ratio). Glp1r and Gcgr were predominantly expressed in arteriolar VSMCs and distal nephron structures of mouse kidneys respectively, whereas Gipr was the predominant of the three transcripts in mouse hearts. Kidney Glp1r and Gcgr and cardiac Gipr mRNA levels were reduced in DMHFD mice and this reduction was negated or attenuated with finerenone. Mechanistically, finerenone attenuated upregulation of the profibrotic growth factor Ccn2 in DMHFD kidneys, whereas recombinant CCN2 downregulated Glp1r and Gcgr in VSMCs and MDCK cells respectively.

**Conclusions:**

Through its anti-fibrotic actions, finerenone reverses Glp1r and Gcgr downregulation in the diabetic kidney. Both finerenone and GLP-1R agonists have proven cardiorenal benefits, whereas receptor co-agonists are approved or under development. The current findings provide preclinical rationale for the combined use of finerenone with the GLP-1R agonist family. They also provide mechanism of action insights into the potential benefit of finerenone in people with diabetes for whom GLP-1R agonists or co-agonists may not be indicated.

**Supplementary Information:**

The online version contains supplementary material available at 10.1186/s13098-024-01525-3.

## Background

The past decade has seen major therapeutic advances transform the clinical care of people living with diabetes, reducing (although not negating) the future risk of kidney and cardiovascular outcomes. Sodium-glucose cotransporter-2 (SGLT2) inhibitors, for instance, have repeatedly shown themselves to reduce cardiorenal events and are now firmly embedded in clinical practice guidelines. More recently, the mineralocorticoid receptor antagonist finerenone has similarly been found to improve cardiovascular [1] and kidney [2] outcomes in people with Type 2 diabetes and chronic kidney disease (CKD), as have glucagon-like peptide-1 (GLP-1) receptor agonists (GLP-1RAs) [3, 4]. The dual GLP-1RA/gastric inhibitory polypeptide receptor (GIPR) agonist tirzepatide has received regulatory authority approval, and triple agonists of GLP-1R, GIPR and the glucagon receptor (GCGR) [5], or dual GLP-1R/GCGR agonists [6] are under development. Early indications from post-hoc analyses of clinical trials [7, 8], or mechanistic studies using preclinical models [9, 10], suggest that these therapies may also ultimately show themselves to have favourable cardiovascular and kidney profiles. Thus, a number of new therapies have recently emerged with proven cardiorenal benefits in people with diabetes, and the future looks bright that further advances are on the horizon. One major unanswered question, however, is how the different pathways affected by different therapies may intersect. Because these treatments were developed in parallel and seemingly unconnectedly, at the time of their clinical trial participation many individuals were not receiving concurrent treatment with other therapies subsequently also found to have kidney or cardiovascular protective benefits. For instance, in the Finerenone in Reducing Kidney Failure and Disease Progression in Diabetic Kidney Disease (FIDELIO-DKD) trial, over 80% of trial participants were neither treated with a GLP-1RA at enrolment nor initiated on a GLP-1RA during study participation [2].

Finerenone is a first in-class, orally administered, nonsteroidal mineralocorticoid receptor antagonist. It attenuates the inflammation and fibrosis propagated by overactivation of the mineralocorticoid receptor in kidney and heart disease [11], and the favourable kidney and cardiovascular outcome trials of finerenone in 2020/2021 [1, 2] led to its approval by the FDA in 2021.

We initiated the present study mindful of the dearth of scholarship attesting to the additive, synergistic or potentially redundant overlap between the molecular processes modified by finerenone and other pathways implicated in kidney and heart disease in diabetes targeted by other therapies. To gain mechanistic insights into this intersection, we chose to administer finerenone to mice in a manner designed to mimic how it is used clinically in patients, late in the course of the disease and in the presence of comorbidity. We evaluated the cardiorenal phenotype of the mice, assessed how this is altered by late initiation of finerenone and determined how, in turn, finerenone affects the cardiorenal expression of *Glp1r*, *Gipr* and *Gcgr*.

## Methods

### Mouse studies

Male C57BL/6J mice (stock 000664) aged ~ 7–8 weeks were obtained from The Jackson Laboratory (Bar Harbor, ME). Mice were fed a high fat diet (HFD) (45% kcal fat, 35% kcal carbohydrate, and 0.05% w/w cholesterol; D12451, Research Diets Inc., New Brunswick, NJ) for 26 weeks. After 12 weeks of HFD feeding, mice received a single intraperitoneal injection of streptozotocin (STZ; 90 mg/kg in 0.1 mol/L sodium citrate, pH 4.5) after a 5 h fast. Diabetes was confirmed by random blood glucose testing one week after STZ injection (OneTouch UltraMini; LifeScan Canada Ltd., Burnaby, British Columbia, Canada) (DMHFD mice). After 26 weeks of HFD feeding, mice were randomized to continue to receive HFD for a further two weeks or were treated with finerenone (HY-111372, MedChemExpress, Monmouth Junction, NJ) in HFD (100 mg/kg diet, 45% kcal fat; D23041405, Research Diets Inc.). Dosing of finerenone was based on that previously reported in [12]. Control mice were fed standard chow throughout the study period and did not receive STZ. At the end of the study period, random blood glucose was determined by OneTouch UltraMini and HbA_1c_ was measured by A1cNow (PTS Diagnostics, Whitestown, IN). Systolic blood pressure was measured using a CODA noninvasive blood pressure system (Kent Scientific, Torrington, CT) [13]. Urine albumin excretion was determined using Albuwell M ELISA (Ethos Biosciences, Newtown Square, PA) after housing mice individually in metabolic cages for 24 h. Transthoracic echocardiography was performed under 1% isoflurane using a high-frequency ultrasound system (Vevo 2100, MS550D transducer, Visual Sonics Inc., Toronto, Ontario, Canada). All experimental procedures adhered to the guidelines of the Canadian Council of Animal Care and were approved by the St. Michael’s Hospital Animal Care Committee.

### Glomerular volume

Glomerular volume was determined on 30 glomerular profiles on H&E stained kidney sections by an investigator masked to the study groups, as previously described [13].

### Glomerulosclerosis index

Glomerulosclerosis index was calculated semi-quantitatively from 30 glomerular profiles on periodic acid-Schiff (PAS)-stained kidney sections by an investigator masked to the study groups, as previously described [14].

### Picrosirius red staining

To estimate kidney and heart interstitial collagen content, tissue sections were stained with picrosirius red and digitized with an Axio Scan.z1 (Carl Zeiss Microscopy, Jena, Germany). The proportional area positively staining red was analysed in a masked manner using Fiji/Image J 1.54f (National Institutes of Health, Bethesda, MD).

### Flow cytometry

Kidney immune cell accumulation was determined by flow cytometry on subsets of five mice per group. For surface marker staining, cells were incubated with the following conjugated anti-mouse antibodies: CD45 PE-Cy7 (catalog no. 103114, lot no. B271123, BioLegend, San Diego, CA), Ly6C BV650 (catalog no. 128035, lot no. B386737, BioLegend), CD11b PE (catalog no. 12-0112-82, lot no. 2460179, Thermo Fisher Scientific, Waltham, MA), F4/80 APC (catalog no. 17-4801-82, lot no. 2555809, Thermo Fisher Scientific), CD11c PE-Dazzle (catalog no. 117348, lot no. B391513, BioLegend), CD4 FITC (catalog no. 11-0042-86, lot no. 2321282, Thermo Fisher Scientific). Samples were analyzed using a Beckman Coulter Life Sciences Cytoflex LX flow cytometer, and the data were processed using FlowJo (v10.6.1., BD Biosciences).

### Myocyte size

Cardiomyocyte cross-sectional area was calculated on 30 myocytes on H&E-stained heart sections by an investigator masked to the study groups, as previously described [15, 16], and based on the method previously described by Frustaci et al. [17].

### Cardiac capillary density

For determination of cardiac capillary density, heart sections were stained with biotin-XX conjugated isolectin GS-IB_4_ (catalog no. I21414; lot no. 1110271; Thermo Fisher Scientific) at 1:500 dilution, followed by VECTASTAIN Elite ABC-HRP kit (catalog no. PK-6100; lot no. 2G0924; Vector Laboratories, Newark, CA). Color development was completed using Dako Liquid DAB + Substrate Chromogen System (K3467 Aligent Technologies Inc., Santa Clara, CA). Stained slides were scanned with an Axio Scan.z1 and analysed using Fiji/ImageJ 1.54f. Capillary density was calculated by automated counting method in at least five randomly selected x20 fields in each section.

### RNAscope in situ hybridization

*Glp1r*, *Gipr* and *Gcgr* transcripts were probed for using RNAscope in situ hybridization (Advanced Cell Diagnostics, Hayward, CA) according to the manufacturer’s instructions [18, 19], with the following probesets: *Glp1r* (catalog no. 418851, lot no. 23285B), *Gipr* (catalog no. 319121, lot no. 23317 A), *Gcgr* (catalog no. 437091, lot no. 23332 A). The bacterial gene *dapB* (catalog no. 310043, lot no. 2017575) served as the negative control. RNAscope puncta were quantified by manual counting in six randomly selected fields in five kidney and heart sections from control mice (x400 magnification). For glomeruli, proximal tubules, interstitial cells and vascular smooth muscle cells, six kidney cortical fields were selected; for kidney distal tubule/collecting ducts, three cortical and three medullary fields were selected; and for heart sections, six myocardial fields were selected.

### Quantitative reverse transcription polymerase chain reaction

Total RNA was isolated using TRIzol reagent (Thermo Fisher Scientific) and cDNA was reverse transcribed from 2 µg RNA using a High-Capacity cDNA Reverse Transcription Kit (Thermo Fisher Scientific). Primer sequences were from Integrated DNA Technologies (Coralville, IA) or Origene (Rockville, MD) and are listed in Supplementary Table 1. SYBR green-based qRT-PCR was performed on a QuantStudio 7 Flex Real-Time PCR System (Thermo Fisher Scientific). Data analysis was performed using the comparative ΔΔC_T_ method.

### Isolation of primary vascular smooth muscle cells (VSMCs)

Primary VSMCs were isolated from the aortas of male C57BL/6J mice aged ~ 7–8 weeks following the protocol described by Hubert et al. [20]. VSMCs were characterized by phase contrast microscopy and by immunostaining for transgelin (also named TAGLN, SM22 and SM22α). For transgelin immunofluorescence, cells were stained with rabbit polyclonal anti-transgelin antibody at 1:200 dilution (catalog no. ab14106, lot no. 1071208-1, Abcam, Waltham, MA) and Alexa Fluor 488 donkey anti-rabbit secondary antibody (catalog no. a-21206, lot no. 2156521, Thermo Fisher Scientific) and mounted with DAPI Fluoromount-G Mounting Medium (catalog no. 17984-24, lot no. 220826 (E2222-RA42), Electron Microscopy Sciences, Hatfield, PA). Recombinant rabbit IgG, monoclonal [EPR25A] diluted 1:200 (catalog no. ab172730, Abcam) served as the negative control. Slides were viewed on an Axio Observer Z1 fluorescence microscope using Zen3.5 (Carl Zeiss Microscopy).

### Cell culture

NRK-49 F cells [21], HK-2 cells [22] and MDCK cells [23, 24] were cultured as previously described. VSMCs and MDCK cells were cultured with 100nmol/L aldosterone [25] (MilliporeSigma) with or without 5µmol/L finerenone [26, 27] for 24 h prior to qRT-PCR. HK-2 cells were incubated in the presence or absence of 100nmol/L aldosterone for 24 h. NRK-49 F cells were treated with or without 100nmol/L aldosterone, in the presence or absence of 5µmol/L, 50µmol/L or 100µmol/L finerenone for 24 h prior to qRT-PCR for *Ccn2* [27, 28]. VSMCs were treated with 100ng/mL recombinant mouse CCN2 [29] (Abx066092, Abbexa, Cambridge, UK) and MDCK cells were treated with 100ng/mL recombinant human CCN2 (CYT-687, ProSpec, East Brunswick, NJ; 93.4% amino acid sequence species similarity) for 24 h prior to qRT-PCR for *Glp1r* and *Gcgr* respectively.

### Statistics

Data are expressed as mean ± S.D. Statistical difference was determined by two-tailed paired or unpaired Student t test for comparison of two groups or Mann-Whitney U test as indicated, by one-way ANOVA followed by Fisher’s least significant difference post hoc test for comparison of three groups or Tukey’s post hoc test for comparison of four groups, unless otherwise stated. All statistical analyses were performed using GraphPad Prism 10 for Mac OS X (GraphPad Software, San Diego, CA). A *P* value < 0.05 was considered statistically significant.

## Results

### Metabolic parameters in DMHFD mice are unaffected by finerenone

**Fig. 1:**
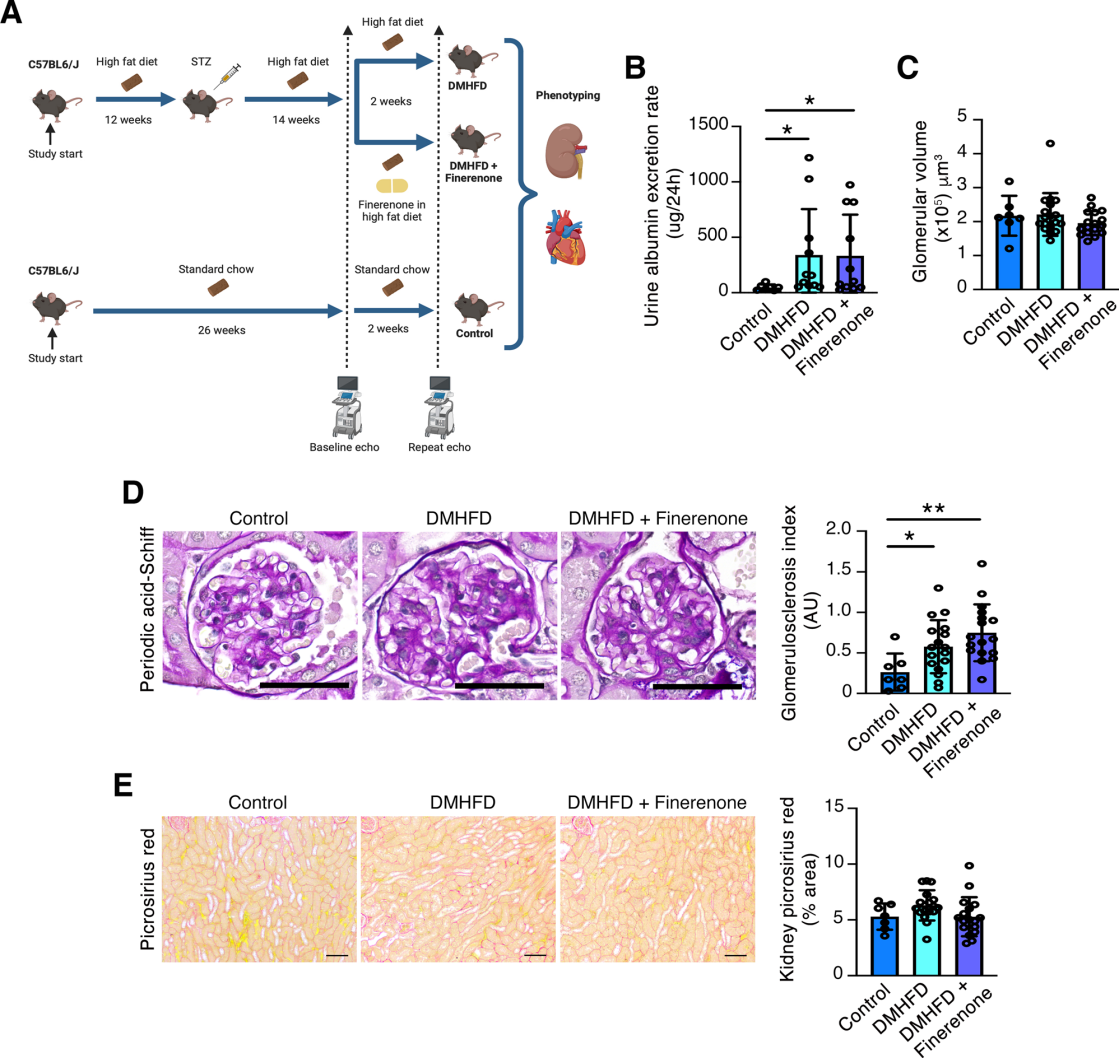
Kidney phenotyping of age-matched control mice (control), diabetic high fat-diet fed mice (DMHFD), or DMHFD mice treated with finerenone (100 mg/kg diet) in high fat diet for the final 2 weeks (DMHFD + Finerenone). (**A**) Study design. (**B**) Urine albumin excretion in control (*n* = 7), DMHFD (*n* = 11) and DMHFD + Finerenone (*n* = 11) mice. (**C**) Glomerular volume determined on H&E stained kidney sections in control (*n* = 7), DMHFD (*n* = 18) and DMHFD + Finerenone (*n* = 17) mice. (**D**) Representative photomicrographs of kidney periodic acid-Schiff staining and quantitation of glomerulosclerosis index in control (*n* = 7), DMHFD (*n* = 17) and DMHFD + Finerenone (*n* = 17) mice. Original magnification x400. Scale bar = 50 μm. (**E**) Representative photomicrographs of kidney picrosirius red staining and quantitation of kidney picrosirius red in control (*n* = 7), DMHFD (*n* = 17) and DMHFD + Finerenone (*n* = 17) mice. Original magnification x100. Scale bar = 100 μm. Values are mean ± S.D. *P < 0.05, **P < 0.01 by one-way ANOVA followed by Fisher’s least significant difference test. Skew distributed data in (**B**) were log transformed before statistical comparison

To explore the transcriptional effects of finerenone in mice with comorbid diabetes, we adopted a reverse translational approach and studied aged mice fed a HFD with STZ-induced hyperglycemia (DMHFD mice). Three groups of mice were studied: age-matched control mice (control), DMHFD mice, and DMHFD mice fed finerenone in HFD for the final two weeks of study (DMHFD + Finerenone). The study design is illustrated in Fig. 1A. Metabolic parameters of the three mice groups are shown in Table 1. There was no difference between control and DMHFD mice in body weight, heart weight, kidney weight, systolic blood pressure, or food consumption, whereas blood glucose and HbA_1c_ were expectedly higher in DMHFD mice than control mice (Table 1). None of these parameters differed significantly between DMHFD mice treated with finerenone for two weeks and DMHFD mice continued on HFD alone, although heart weight corrected for tibial length was numerically lower in finerenone-treated mice (Table 1).

**Table 1:** Metabolic parameters in control mice and DMHFD mice fed either high fat diet or finerenone in high fat diet

	Control	DMHFD	DMHFD + Finerenone
*n*	7	18	17
Body weight (g)	36±2	39±9	34±7
Heart weight (mg)	161±17	158±20	145±18
Heart weight: tibial length (mg/mm)	8.3±0.7	8.2±1.0	7.6±0.9
Mean kidney weight (mg)	206±17	219±53	199±33
Mean kidney weight: body weight (%)	0.57±0.04	0.62±0.30	0.60±0.16
Systolic blood pressure (mmHg)	100±10	94±7 (*n* = 17)	97±7
Random blood glucose (mmol/L)	8.9±1.3	19.1±9.7^a^	23.8±8.9^b^
HbA_1c_ (%)	4.4±0.2	7.1±2.9^a^ (*n* = 17)	8.0±2.5^c^
HbA_1c_ (mmol/mol)	24.7±2.0	55.6±31.5^a^ (*n* = 17)	63.7±27.5^c^
Chow intake (g/day)	3.5±0.8	2.9±1.2	2.9±1.4

Values are mean ± S.D. Chow intake determined while mice housed individually in metabolic cages. ^a^*P*<0.05, ^b^*P*<0.001, ^c^*P*<0.01 vs. control by one-way ANOVA followed by Fisher’s least significant difference test

### The kidney phenotype of DMHFD mice is characterized by albuminuria, mesangial matrix expansion and *Ifng* upregulation

**Fig. 2:**
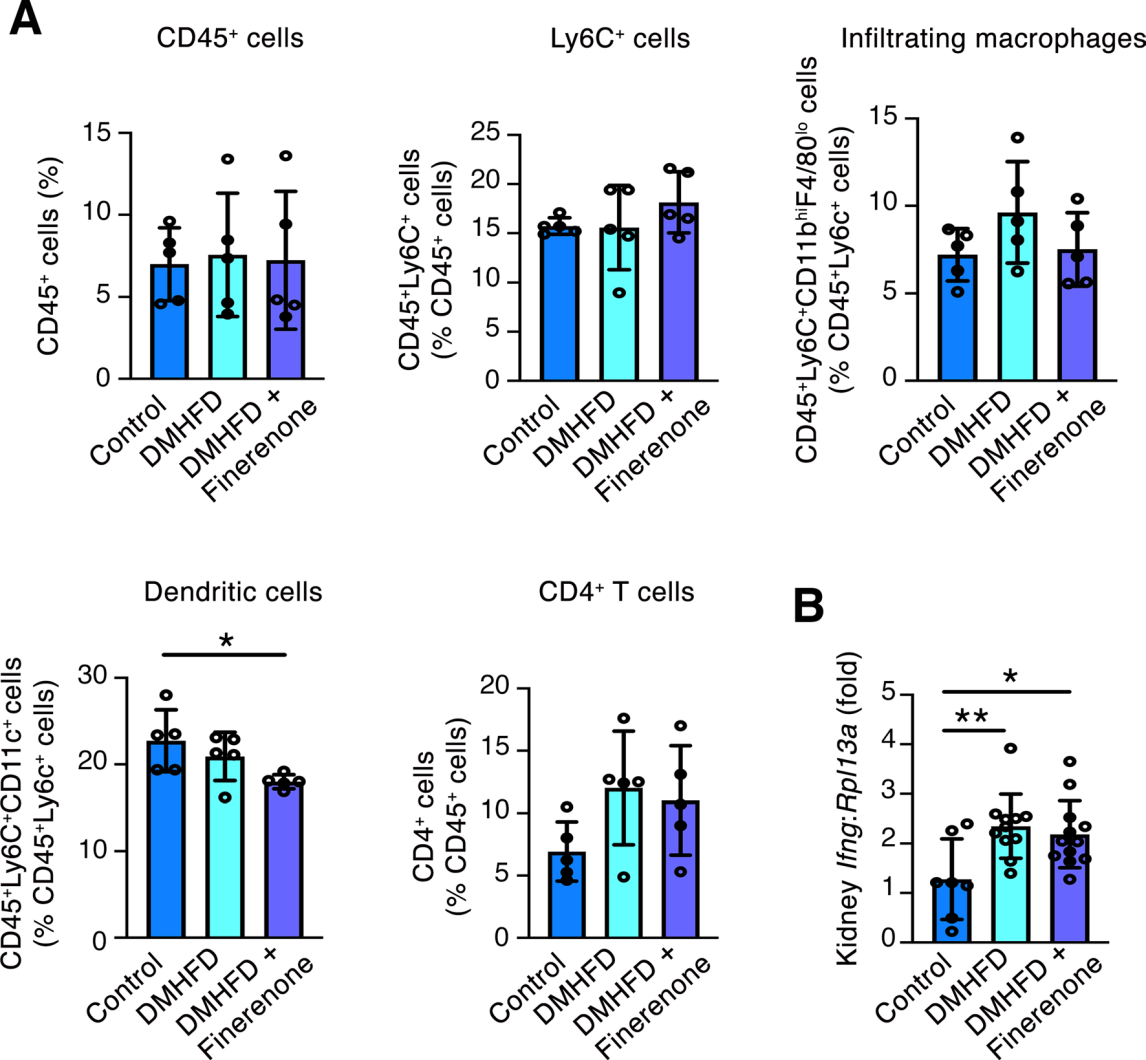
Immune cell populations and *Ifng* mRNA levels in the kidneys of age-matched control mice (control), diabetic high fat-diet fed mice (DMHFD), or DMHFD mice treated with finerenone (100 mg/kg diet) in high fat diet for the final 2 weeks (DMHFD + Finerenone). (**A**) Flow cytometry for CD45^+^ cells, CD45^+^Ly6C^+^ cells, infiltrating macrophages, dendritic cells and CD4^+^ T cells (*n* = 5/group). (**B**) qRT-PCR for *Ifng* in the kidneys of control (*n* = 7), DMHFD (*n* = 11) and DMHFD + Finerenone (*n* = 12) mice. Values are mean ± S.D. *P < 0.05, **P < 0.01 by one-way ANOVA followed by Fisher’s least significant difference test

Urine albumin excretion was increased in DMHFD mice compared to controls and was unaffected by finerenone (Fig. 1B), whereas glomerular volume (determined on H&E stained kidney sections) was unaltered across the groups (Fig. 1C). Mesangial matrix accumulation, assessed using a semiquantitative glomerulosclerosis index on PAS-stained kidney sections was increased in DMHFD mice and was unaffected by two-week treatment with finerenone (Fig. 1D). Interstitial fibrosis (as determined by picrosirius red staining) did not differ between the three groups (Fig. 1E). To determine whether immune cell populations differed in the kidneys of control and DMHFD mice or with finerenone treatment, flow cytometry was performed to enumerate five populations of immune cells in a subset of animals (Supplementary Fig. 1): CD45^+^ cells, Ly6C^+^ cells (CD45^+^Ly6C^+^), infiltrating macrophages (CD45^+^Ly6C^+^CD11b^hi^F4/80^lo^), dendritic cells (CD45^+^Ly6C^+^CD11c^+^) and CD4^+^ T cells. Infiltrating macrophages and CD4^+^ T cells tended to be higher in the kidneys of DMHFD mice compared to controls, but this did not achieve significance on multiple groups comparison, whereas dendritic cells were lower in DMHFD + Finerenone kidneys than controls (Fig. 2A). Consistent with a tendency to CD4^+^ T cell and macrophage accumulation in DMHFD kidneys, mRNA levels of the type II interferon *Ifng* were increased in DMHFD kidneys and were unaffected by two-week treatment with finerenone (Fig. 2B).

### Finerenone corrects decreased E/A ratio and increased myocyte size in DMHFD mice

**Fig. 3:**
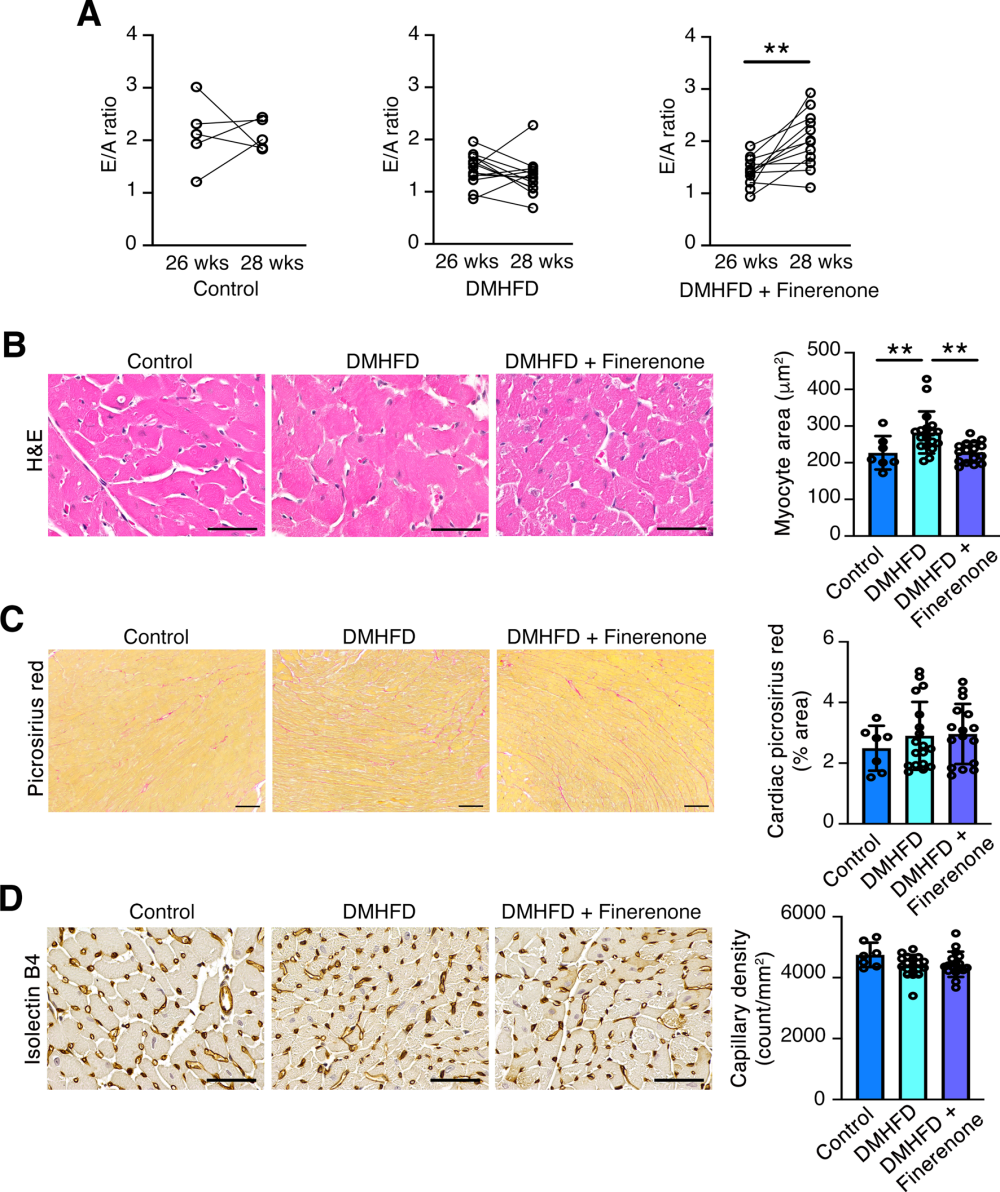
E/A ratio and cardiac histopathology in age-matched control mice (control), diabetic high fat-diet fed mice (DMHFD), or DMHFD mice treated with finerenone (100 mg/kg diet) in high fat diet for the final 2 weeks (DMHFD + Finerenone). (**A**) E/A ratio before treatment (26 weeks (wks)) and two weeks after finerenone or vehicle (28 wks). Control (*n* = 5), DMHFD (*n* = 13), DMHFD + Finerenone (*n* = 12). (**B**) Representative H&E stained heart sections and cardiomyocyte cross sectional area determined on H&E-stained kidney sections in control (*n* = 7), DMHFD (*n* = 18), DMHFD + Finerenone (*n* = 16). Original magnification x400. Scale bar = 50 μm. (**C**) Representative photomicrographs of cardiac picrosirius red staining and quantitation of cardiac picrosirius red in control (*n* = 7), DMHFD (*n* = 18) and DMHFD + Finerenone (*n* = 16) mice. Original magnification x100. Scale bar = 100 μm. (**D**) Representative photomicrographs of isolectin B4 staining and quantitation of cardiac capillary density in control (*n* = 7), DMHFD (*n* = 18) and DMHFD + Finerenone (*n* = 17) mice. Original magnification x400. Scale bar = 50 μm. Values are mean ± S.D. **P < 0.01 by two-tailed paired Student t test (**A**) or by one-way ANOVA followed by Fisher’s least significant difference test (**B**)

Cardiac function was assessed by M-mode echocardiography in control and DMHFD mice prior to starting finerenone feeding (or HFD/normal chow) and two weeks later (Table 2). There was no difference in left ventricular (LV) mass, chamber dimensions, wall thickness, cardiac output or stroke volume between the three groups either at baseline or after two weeks (Table 2). Ejection fraction and fractional shortening were statistically lower in vehicle-treated DMHFD mice than control mice at baseline, but not on repeat testing two weeks later (Table 2). E/A ratio from both echocardiograms was determinable in 30 mice. E/A ratio at 26 weeks and prior to randomization to finerenone-treatment was lower in DMHFD mice than controls (E/A ratio, control (*n* = 5) 2.1±0.7, DMHFD (*n* = 25) 1.4±0.3, *P* < 0.05 Mann-Whitney U test). Despite the short duration of treatment, E/A ratio increased in DMHFD mice randomized to receive finerenone for two weeks, whereas it remained unchanged in the other two groups (Fig. 3A). Histologically, myocyte size was increased in vehicle-fed DMHFD mice but not those treated with finerenone (Fig. 3B), whereas neither interstitial fibrosis (as determined by picrosirius red staining) nor cardiac capillary density (determined following isolectin B4 staining) were altered in any of the three groups (Fig. 3C and D).

**Table 2:** Cardiac parameters in control mice and DMHFD mice fed either high fat diet or finerenone in high fat diet

	Before treatment (26 weeks)			After treatment (28 weeks)		
	Control	DMHFD	DMHFD + Finerenone	Control	DMHFD	DMHFD + Finerenone
*n*	7	18	17	7	18	17
Heart rate (bpm)	403±57	359±45	378±62	379±46	381±53	389±49
Left ventricular mass (mg)	134±21	134±23	121±16	148±25	136±28	130±19
Ejection fraction (%)	65±10	56±7^a^	61±8	59±14	60±7	55±10
Fractional shortening (%)	36±7	29±5^a^	32±5	32±2	32±5	28±6
Cardiac output (ml/min)	20±5	16±4	16±3	18±4	15±4	16±3
Stroke volume (µl)	50±13	45±9	43±8	48±12	39±8	41±7
LVDs (mm)	2.7±0.4	3.0±0.4	2.7±0.4	2.9±0.7	2.7±0.4	2.9±0.4
LVDd (mm)	4.1±0.3	4.2±0.3	4.0±0.4	4.3±0.5	3.9±0.4	4.1±0.3
LVESV (µl)	27±9	36±11	29±10	35±16	28±11	34±11
LVEDV (µl)	77±14	81±16	72±15	83±22	67±17	76±12
LVAWT (mm)	0.99±0.13	0.95±0.15	0.93±0.13	1.02±0.13	0.97±0.15	1.00±0.18
LVPWT (mm)	0.90±0.13	0.82±0.11	0.85±0.13	0.91±0.10	0.92±0.15	0.84±0.13

bpm = beats per minute, LVDs = left ventricular internal diameter at systole, LVDd = left ventricular internal diameter at diastole, LVESV = left ventricular end systolic volume, LVEDV = left ventricular end diastolic volume, LVAWT = left ventricular anterior wall thickness, LVPWT = left ventricular posterior wall thickness. Values are mean ± S.D. ^a^*P*<0.05, vs. control by one-way ANOVA followed by Fisher’s least significant difference test

### Kidney *Glp1r* is mainly expressed in arteriolar smooth muscle cells and *Gcgr* is mainly expressed in the distal nephron, whereas *Gipr* is the predominant of the three transcripts in mouse hearts

**Fig. 4:**
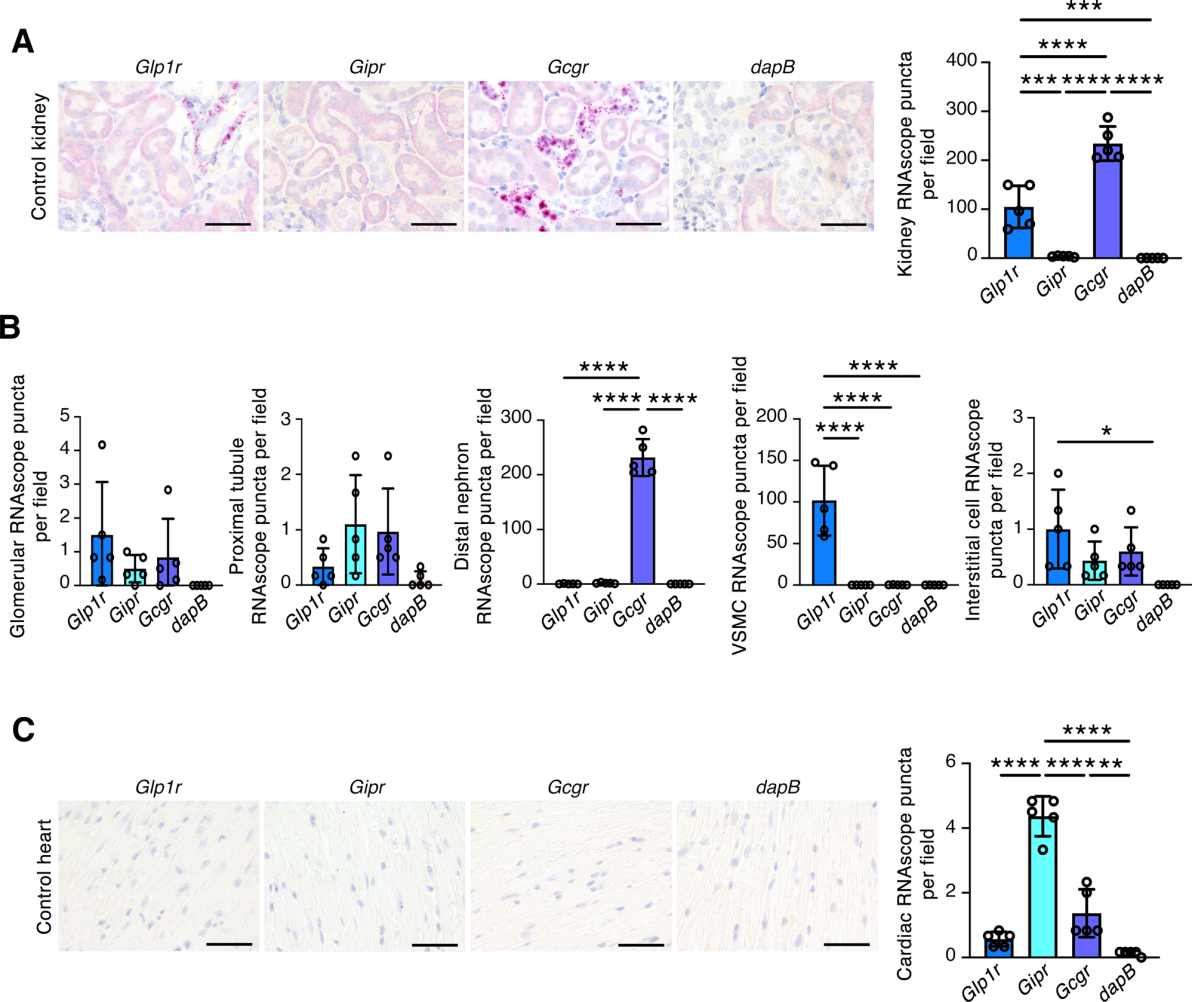
RNAscope in situ hybridization for *Glp1r*, *Gipr* and *Gcgr* in mouse kidneys and hearts. *dapB* = negative control. (**A**) Representative kidney sections and quantitation of RNAscope puncta per x400 magnification field in control mouse kidneys (*n* = 5/transcript). Original magnification x400. Scale bar = 50 μm. (**B**) Quantitation of RNAscope puncta per x400 magnification field in glomeruli, proximal tubule cells, distal nephron cells, vascular smooth muscle cells (VSMCs) and interstitial cells in mouse kidney sections (*n* = 5/transcript). (**C**) Representative cardiac cross sections and mean RNAscope puncta for *Glp1r*, *Gipr*, *Gcgr* and *dapB* per x400 magnification field in control mouse hearts (*n* = 5/transcript). Original magnification x400. Scale bar = 50 μm. Values are mean ± S.D. *P < 0.05, **P < 0.01, ***P < 0.001, ****P < 0.0001 by one-way ANOVA followed by Tukey’s post-hoc test

Next, we turned our attention to the patterns of expression of kidney and heart *Glp1r*, *Gipr* and *Gcgr*. In mouse kidneys, *Glp1r* transcripts were readily detectable in smooth muscle cells of arterioles and were present but in low abundance in interstitial cells; *Gcgr* transcripts were abundant in distal nephron structures; whereas *Gipr* RNAscope puncta did not exceed background levels (Fig. 4A and B). In mouse hearts, *Gipr* was the predominant of the three transcripts, *Gcgr* transcripts were quantifiable but of low abundance, whereas the number of *Glp1r* RNAscope puncta did not differ significantly from the negative control, *dapB* (Fig. 4C). There was no difference in the site-specific expression of any of the transcripts between control and DMHFD mice or between DMHFD mice treated with vehicle and finerenone (Supplementary Figs. 2 and 3).

### Finerenone attenuates kidney *Glp1r* and *Gcgr* and cardiac *Gipr* downregulation in DMHFD mice

**Fig. 5:**
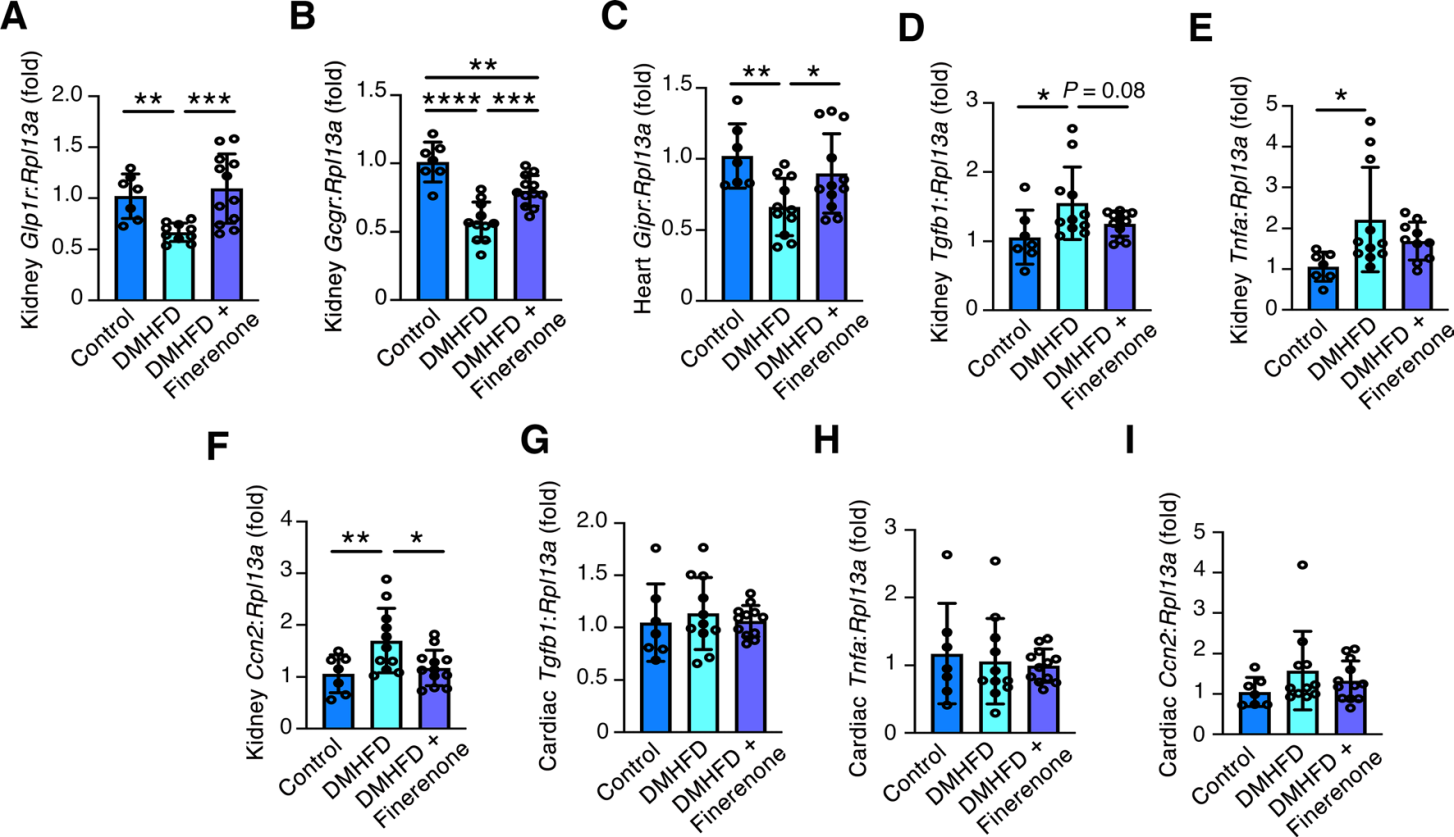
Finerenone negates kidney *Glp1r* and *Gcgr* and cardiac *Gipr* downregulation in mice with comorbid diabetes. qRT-PCR for kidney *Glp1r*, kidney *Gcgr*, cardiac *Gipr*, and kidney and heart *Tgfb1*, *Tnfa* and *Ccn2* in age-matched control mice (control), diabetic high fat-diet fed mice (DMHFD), or DMHFD mice treated with finerenone (100 mg/kg diet) in high fat diet for the final 2 weeks (DMHFD + Finerenone). Kidney *Glp1r* (**A**), kidney *Gcgr* (**B**), heart *Gipr* (**C**), kidney *Tgfb1* (**D**), kidney *Tnfa* (**E**), kidney *Ccn2* (**F**), heart *Tgfb1* (**G**), heart *Tnfa* (**H**), heart *Ccn2* (**I**) in control (*n* = 7), DMHFD (*n* = 11), DMHFD + Finerenone (*n* = 12); except (**A**) DMHFD (*n* = 10), and (**E**) DMHFD + Finerenone (*n* = 10). Values are mean ± S.D. *P < 0.05, **P < 0.01, ***P < 0.001, ****P < 0.0001 by one-way ANOVA followed by Fisher’s least significant difference test

Having identified enumerable *Glp1r* and *Gcgr* transcripts in mouse kidneys and *Gipr* transcripts in mouse hearts, we focused our next experiments on discerning relative mRNA levels of these genes in the three mouse groups. By qRT-PCR, kidney *Glp1r* and *Gcgr* and cardiac *Gipr* were lower in each case in DMHFD mice than controls, with this reduction being attenuated or negated with finerenone (Fig. 5A-C). In considering potential mechanisms for transcript downregulation, we perfomed qRT-PCR for *Tgfb1*, *Tnfa* and *Ccn2*, three genes well-established for their roles in the development of diabetes complications. *Tgfb1*, *Tnfa* and *Ccn2* were each increased in DMHFD kidneys in comparison to controls (Fig. 5D-F). *Tgfb1* and *Tnfa* mRNA levels tended to be lower in the kidneys of DMHFD mice treated with finerenone than in DMHFD mice continued on a HFD, although this did not achieve statistical significance (Fig. 5D and E). In contrast, finerenone negated the increase in *Ccn2* in DMHFD mouse kidneys (Fig. 5F). Cardiac *Tgfb1*, *Tnfa* and *Ccn2* mRNA levels did not differ between the three groups (Fig. 5G-I).

### Aldosterone upregulates *CCN2* in cultured kidney cells and CCN2 downregulates VSMC *Glp1r* and MDCK cell *Gcgr*

**Fig. 6:**
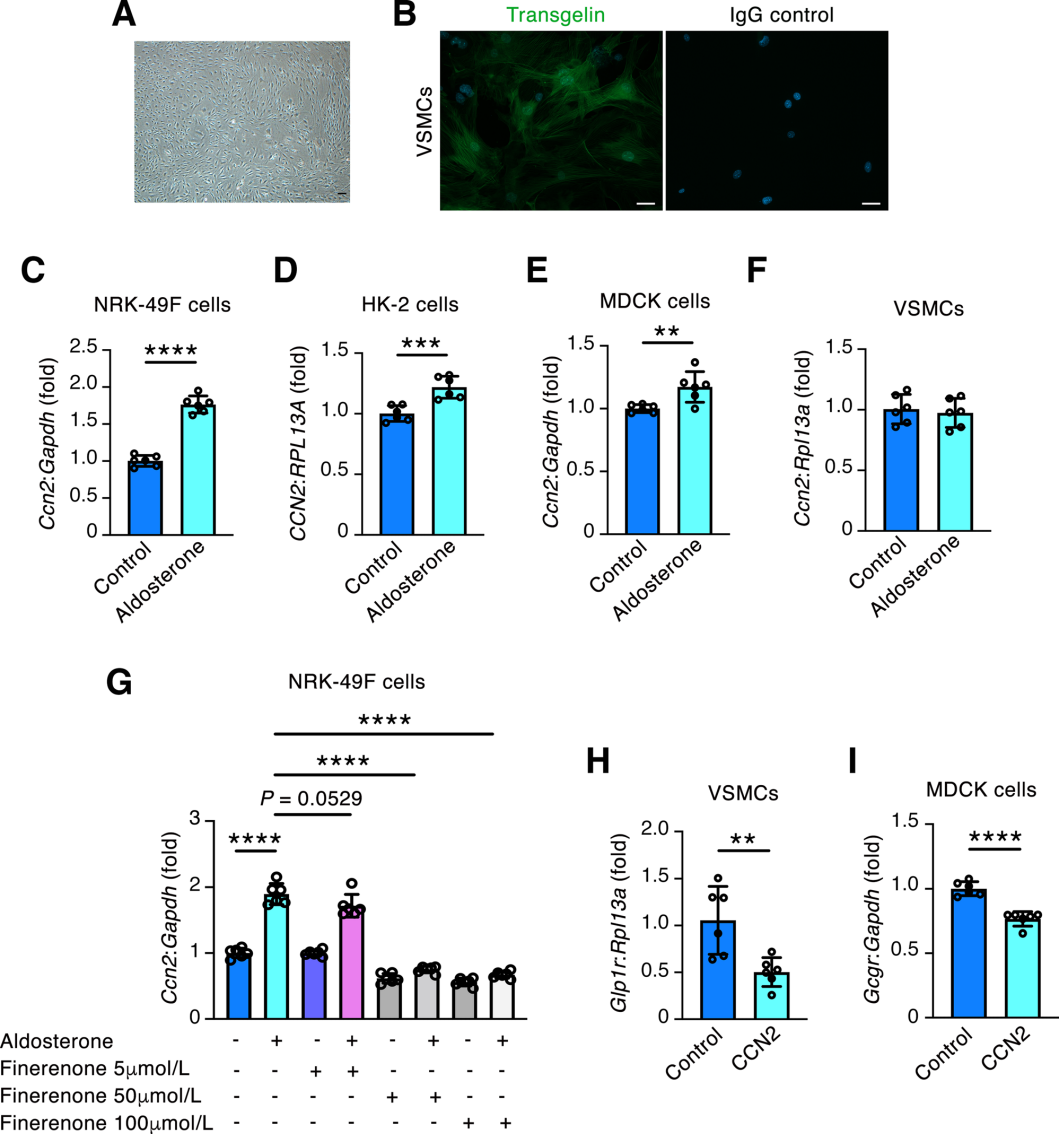
Aldosterone upregulates *CCN2* in NRK-49 F cells, HK-2 cells and MDCK cells, and CCN2 downregulates *Glp1r* in mouse vascular smooth muscle cells (VSMCs) and *Gcgr* in MDCK cells. (**A** and **B**) Characterization of primary cultured VSMCs. (**A**) Phase-contrast microscopy image of VSMCs. Original magnification x100. Scale bar = 50 μm. (**B**) Immunofluorescence staining for the smooth muscle cell marker transgelin. Scale bar = 50 μm. Experiment performed in duplicate. (**C**-**F**) qRT-PCR for *CCN2* in NRK-49 F cells (**C**), HK-2 cells (**D**), MDCK cells (**E**) and VSMCs (**F**) treated under control conditions, or with 100nmol/L aldosterone for 24 h (*n* = 6/condition). (**G**) qRT-PCR for *Ccn2* in NRK-49 F cells treated in the presence or absence of 100nmol/L aldosterone with or without finerenone (5µmol/L, 50µmol/L, or 100µmol/L) (*n* = 6/condition). (**H** and **I**) qRT-PCR for *Glp1r* in VSMCs (**H**) or *Gcgr* in MDCK cells (**I**) under control conditions, or with 100ng/mL recombinant CCN2 for 24 h (*n* = 6/condition). Values are mean ± S.D. **P < 0.01, ***P < 0.001, ****P < 0.0001 by unpaired two-tailed Student t test (**C**, **D**, **E**, **F**, **H**, **I**) or one-way ANOVA with Sidak’s post-test (**G**)

Having observed that finerenone attenuated *Glp1r* and *Gcgr* downregulation and fibrotic growth factor upregulation in DMHFD mouse kidneys, lastly we sought to determine whether there was a causative relationship between these transcript changes. We first isolated VSMCs from the aortas of mice. These cells exhibited a typical spindle-shaped morphology under phase-contrast microscopy (Fig. 6A), and they were characterized by their expression of transgelin by fluorescence microscopy (Fig. 6B). Aldosterone-treatment numerically (but non-significantly) lowered *Glp1r* levels in VSMCs, whereas finerenone blocked this effect (Supplementary Fig. 4A). In contrast, there was an unexpected small but statistically significant increase in *Gcgr* levels in MDCK canine distal tubule lineage cells treated with aldosterone (Supplementary Fig. 4B). Next, we treated NRK-49 F rat kidney fibroblasts, HK-2 human proximal tubule lineage cells, MDCK cells and VSMCs with aldosterone and observed an increase in *CCN2* mRNA levels in NRK-49 F cells (Fig. 6C), HK-2 cells (Fig. 6D) and MDCK cells (Fig. 6E) (but not VSMCs (Fig. 6F)), this increase being most prominent in NRK-49 F cells (Fig. 6C). We repeated the experiment in NRK-49 F cells and observed that finerenone dose-dependently prevented the increase in *Ccn2* mRNA levels induced by aldosterone (Fig. 6G). Lastly, to determine whether CCN2 in turn can regulate *Glp1r* and *Gcgr* expression, we treated VSMCs and MDCK cells with recombinant CCN2 observing a diminution in *Glp1r* and *Gcgr* mRNA in VSMCs and MDCK cells respectively (Fig. 6H and I).

## Discussion

The proliferation of recent positive clinical trials reporting cardiovascular and kidney protective benefits of new therapies has been good news for people living with diabetes. However, this rate of clinical development has outpaced the preclinical insights that would have historically underpinned the initiation of such trials. Here, we performed a study in experimental mice and cultured cells to disentangle how pathways affected by some new therapies may interact. We observed that finerenone prevents downregulation of kidney *Glp1r* and *Gcgr* and cardiac *Gipr* in mice with comorbid diabetes, which in the case of kidney *Glp1r* and *Gcgr* may be due to the actions of finerenone in attenuating fibrotic growth factor overelaboration. The findings provide preclinical evidence for an intersection between the biological pathways affected by finerenone and GLP-1R (co-)agonists. They also provide mechanistic rationale for the use of finerenone in people who may be ineligible to receive treatment with GLP-1R (co-)agonists, and for those receiving GLP-1R (co-)agonist treatment who would benefit from additional kidney protection.

We chose to study DMHFD mice because of questions that have been posed as to the ability of monogenic models of diabetes to recapitulate the complex, progressive and multifactorial nature of diabetes as it occurs in most patients [30]. We have taken this approach before to study the effects of dipeptidyl peptidase-4 (DPP-4) inhibition on the heart [31, 32]; and the effects of renin angiotensin system blockade on the expression of angiotensin-converting enzyme 2 (ACE2) and transmembrane serine protease 2 (TMPRSS2) in the heart and kidney [33]. We adopted a similar approach to explore the effects of finerenone on *Glp1r*, *Gipr* and *Gcgr*, first establishing the cardiorenal phenotype of DMHFD mice. Our phenotyping studies indicated that, despite six months of follow-up, the end organ injury in DMHFD mice is relatively subtle, reflected by mild albuminuria, mild mesangial matrix accumulation, and a diminished E/A ratio indicative of diastolic dysfunction.

We initiated our study after the reporting of the FIDELIO-DKD and Finerenone in Reducing Cardiovasular Morbidity and Mortality in Diabetic Kidney Disease (FIGARO-DKD) clinical trials [1, 2]. Our goal was not to recapitulate these clinical trial findings in experimental mice, but rather to determine whether finerenone may affect the expression levels of GLP-1R, GIPR and/or GCGR that are being targeted by approved therapies or those under development. To help us distinguish cause from consequence, we thus administered finerenone for just two weeks, after DMHFD mice had been aged. We did not see an effect of finerenone on the kidney phenotype of DMHFD mice. We posit that this is because of the short duration of treatment and late-stage intervention with finerenone rather than the dosing. Finerenone dosing was selected based on a previous publication [12]. Given the body weight and food consumption of the mice in this study, finerenone dosed at 100 mg/kg chow equates to a dosing of finerenone of approximately 9 mg/kg/day, in a similar range to that used in other mouse studies [34, 35]. Consistent with a pharmacological effect of finerenone, however, we did unexpectedly observe a reversal of the E/A ratio diminution in DMHFD mice, alongside a normalization of myocyte size. These rapid improvements are aligned with a previous study that reported improvements in left ventricular end-diastolic pressure volume relationship (EDPVR; another marker of diastolic dysfunction) evident within seven days of treatment of Zucker *fa*/*fa* rats with finerenone [36]. In that study, the authors attributed the improvement in cardiac function to increased myocardial perfusion and reduced myocardial reactive oxygen species with finerenone [36]. Whereas a two-week treatment with finerenone did not affect kidney structure or function in DMHFD mice, it did result in changes in gene expression. First, both *Glp1r* and *Gcgr* mRNA levels were reduced in DMHFD mouse kidneys in comparison to controls. Second, this diminution was negated or attenuated with finerenone. Likewise in mouse hearts, *Gipr* which was the predominant of the three transcripts was downregulated in DMHFD mice and this downregulation was negated by finerenone treatment.

Preclinical and clinical studies have now firmly established the kidney protective benefits of GLP-1RAs [4, 37], although whether these benefits are direct or indirect remains to be established [37]. Hyperglycemia has previously been reported to induce GLP-1R downregulation in pancreatic islets [38], and vascular GLP-1R is reduced in humans with obesity [39]. In mouse kidneys, we observed *Glp1r* mRNA to be expressed almost exclusively in arteriolar VSMCs, consistent with validated antibody studies in monkeys and humans [40]. It has been suggested that activation of GLP-1R on afferent arteriolar VSMCs increases renal blood flow [41]. In other vascular beds, activation of GLP-1R has been reported to attenuate VSMC proliferation [42–44], enhance mitochondrial metabolism [45], and promote VSMC re-differentiation [46]. In sum, the expression of *Glp1r* in kidney VSMCs and its downregulation in the kidneys of DMHFD mice is consistent with reports from other models or clinical settings. However, whereas GLP-1 has been reported to have favorable effects on VSMC homeostasis, the contribution of these effects to improved kidney outcomes has not been established in this or previous studies.

Whereas, the expression and action of GLP-1R in the kidney have been under scrutiny for a number of years now, the actions of GCGR in the kidney are only beginning to emerge. Aligned with the present findings, prior studies have indicated that *Gcgr* is expressed in the kidney predominantly in the thick ascending loop of Henle, distal convoluted tubule, connecting tubule and collecting duct [47–49]. A recent study employing constitutive and conditional knockout mice established that the kidney GCGR plays an essential role in renal and systemic homeostasis [9]. In the context of those elegant recent studies [9], our observations raise the hypothesis that preservation of kidney GCGR levels may contribute to the established kidney protective benefits of finerenone. That being said, this hypothesis has not been tested in the design of the current experiments, our findings only allowing us to conclude that finerenone treatment was accompanied by a restoration of kidney *Gcgr* mRNA in the kidneys of DMHFD mice.

The actions of GIPR in cardiac myocytes are incompletely resolved, with both detrimental and beneficial effects reported in the literature. For instance, genetic knockout of *Gipr* decreased LV remodeling and improved survival of mice after experimental myocardial infarction [10], whereas conversely human GIP has been reported to attenuate angiotensin II-induced cardiac hypertrophy and fibrosis [50]. In our own previous experiments, we observed no effects of recombinant GIP on mouse hearts perfused ex vivo [31]. A prespecified meta-analysis of the clinical development program for the dual GIP/GLP-1 receptor co-agonist tirzepatide concluded that tirzepatide did not increase major cardiovascular events in participants with Type 2 diabetes [51]. The dedicated Study of Tirzepatide (LY3298176) Compared with Dulaglutide on Major Cardiovascular Events in Participants With Type 2 Diabetes (SURPASS-CVOT) is scheduled to complete in 2025. However, like with GLP-1RAs, even if this trial demonstrates benefit it will not be possible to discern how much of this benefit is due to direct actions of the co-agonist on heart (or kidney) cells versus indirect effects through improvement in adiposity and glycemia, and their sequelae.

To explore potential mechanisms by which finerenone may normalize transcript downregulation, we chose to focus on kidney *Glp1r* and *Gcgr* given (i) the uncertainty as to the roles of GIPR in the heart; and (ii) the inverse change we observed in fibrotic transcript expression with finerenone in DMHFD kidneys but not mouse hearts. Relative hyperaldosteronism is common in CKD and finerenone’s known mechanism of action is through antagonism of the mineralocorticoid receptor [52]. In the present study, we observed that finerenone normalized upregulation of *Ccn2* (also called connective tissue growth factor, CTGF) in DMHFD kidneys, which itself has a well established role in the pathogenesis of kidney disease in diabetes and in kidney function decline. We also observed that aldosterone induces CCN2 in several kidney cell-types, most notably fibroblasts, and that CCN2, in turn, downregulates VSMC *Glp1r* and MDCK cell *Gcgr*. Thus, *Glp1r* and *Gcgr* downregulation are caused by the paracrine actions of CCN2 (and potentially other growth factors) in diabetic kidney disease. Finerenone restores *Glp1r* and *Gcgr* expression in VSMCs and distal nephron cells respectively, through its anti-fibrotic effects on CCN2-producing kidney cells. Our findings are consistent with findings of the recent report of the effects of knockout of GCGR in the kidney [9], which described that, in MDCK cells, knockdown of *Gcgr* augmented fibrotic gene expression; whereas recombinant TGF-β diminished MDCK cell *Gcgr* expression [9]. Accordingly, it is likely that there are several paracrine regulators of *Glp1r* and *Gcgr* expression in the kidney, not solely CCN2 which was tested in the present study. Furthermore, the biology of CCN2 itself is complex, the protein lacking a unique receptor. Rather, CCN2 exerts its effects through several different mechanisms including acting as an extracellular or intracellular adapter protein; competitively binding to heparan sulfate proteoglycans; blocking matrix binding sites; and binding to cell surface receptors, thus potentially activating numerous different signaling pathways that may vary according to the biological context [53]. However, the specific molecular pathways that are involved in the regulatory effect of CCN2 on *Glp1r* and *Gcgr* expression have not been elucidated.

The present study has additional limitations that warrant particular emphasis. Firstly, we did not seek to determine whether finerenone would attenuate cardiorenal dysfunction in experimental mice, this property already having been proven in humans [1, 2]. Furthermore, our focus on short-term gene expression changes, allows us to distinguish the transcriptional effects of mineralocorticoid receptor antagonism from secondary effects occurring as a consequence of improved kidney health. Secondly, we did not directly address the question as to whether finerenone and GLP-1R (co-)agonists have additive or synergistic benefits when used in combination. Such combination studies should be the subject of future clinical and preclinical experimentation. Thirdly, whereas we were able to tease out the mechanism by which finerenone restores *Glp1r* and *Gcgr* expression in the diabetic kidney, the mechanism for *Gipr* normalization in the diabetic heart remains unresolved given that cardiac fibrosis was unaltered in DMHFD mice. Fourthly, through a non-reductionist lens, whereas our findings indicate that the repression of CCN2 upregulation may play a role in the normalization of kidney *Glp1r* and *Gcgr* with finerenone, we do not propose that this is necessarily the sole mechanism. As already stated, other growth factors or paracrine mediators may also be implicated. Furthermore, we cannot exclude a direct effect of finerenone on *Glp1r* and *Gcgr* expressing cells themselves. For instance, at least in VSMCs, *Glp1r* mRNA levels were higher with finerenone in aldosterone-treated cells. Lastly, because of the well-documented challenges of detecting GLP-1R protein in tissue samples [54], we focused our experiments on the detection of mRNA changes by in situ hybridization and qRT-PCR. Accordingly, we have not demonstrated that the mRNA changes that we observed are paralleled by similar shifts in protein abundance. These limitations notwithstanding, the contribution of the present study is the drawing of a mechanistic link between the actions of different therapies or biological pathways that have been proven to improve kidney and heart outcomes. Specifically, that finerenone may normalize *Glp1r* and *Gcgr* mRNA levels in the kidney by regulating the expression of pro-fibrotic factors functioning in a paracrine capacity.

## Conclusion

In summary, finerenone normalizes *Glp1r* and *Gcgr* that are downregulated in the kidneys of mice with comorbid diabetes, likely through its anti-fibrotic effects on neighbouring cells. The findings lay the groundwork for the design of future studies that could test the combined effects of finerenone and GLP-1R (co-)agonists, and they provide additional rationale for the use of finerenone in at-risk individuals in whom GLP-1 based therapies cannot be used.

## Electronic supplementary material

Below is the link to the electronic supplementary material.

Supplementary Material 1

## Data Availability

Data are available from the corresponding author upon reasonable request.

## Abbreviations

GLP-1: Glucagon-like peptide-1
GIP: Gastric inhibitory polypeptide
GLP-1R: Glucagon-like peptide-1 receptor
GIPR: Gastric inhibitory polypeptide receptor
GCGR: Glucagon receptor
VSMCs: Vascular smooth muscle cells
NRK: Normal rat kidney
HK: Human kidney
MDCK: Madin-Darby canine kidney
DMHFD: Diabetic high fat diet-fed
CCN2: Cellular communication network 2
GLP-1RA: GLP-1R agonist
SGLT2: Sodium-glucose cotransporter-2
CKD: Chronic kidney disease
HFD: High fat diet
STZ: Streptozotocin
DPP-4: Dipeptidyl peptidase-4 (DPP-4)
ACE2: Angiotensin-converting enzyme 2
TMPRSS2: Transmembrane serine protease 2 (TMPRSS2)
EDVPR: End-diastolic pressure volume relationship
CTGF: Connective tissue growth factor
