# Supplementary material for: Finerenone attenuates downregulation of the kidney GLP-1 receptor and glucagon receptor and cardiac GIP receptor in mice with comorbid diabetes
Source: Diabetol Metab Syndr. 2024 Nov 24;16:283. doi: 10.1186/s13098-024-01525-3 (PMC11587750; doi:10.1186/s13098-024-01525-3)
Supplement: Supplementary file 1 — Supplementary Material 1 [file 13098_2024_1525_MOESM1_ESM.pdf]

SUPPLEMENTARY MATERIAL

Supplementary Table 1. Primers and primer sequences.

Species	Gene name	Forward primer sequence (5'->3')	Reverse primer sequence (5'->3')
Mus musculus	*Rpl13a*	GCTCTCAAGGTTGTTCGGCTGA	AGATCTGCTTCTTCTTCCGATA
Mus musculus	*Ifng*	AAAGAGATAATCTGGCTCTGC	GCTCTGAGACAATGAACGCT
Mus musculus	*Glp1r*	OriGene (MP205478)	
Mus musculus	*Gcgr*	ACATCATTCACCTTCTTGTGG	GCCAGCGAGGTCTCCATAG
Mus musculus	*Gipr*	OriGene (MP205265)	
Mus musculus	*Tgfb1*	GCTGCGCTTGCAGAGATTAA	GTAACGCCAGGAATTGTTGCTA
Mus musculus	*Tnfa*	TGATCGGTCCCCAAAGGGAT	TGTCTTTGAGATCCATGCCGT
Mus musculus	*Ccn2*	AAGCTGACCTGGAGGAAAACA	TGCAGCCAGAAAGCTCAAAC
Rattus norvegicus	*Gapdh*	GAACGGGAAGCTCACTGG	GCCTGCTTCACCACCTTCT
Rattus norvegicus	*Ccn2*	CCCGATGGCGAGATCATGAA	TGTCCCTTACTCCCTGGCT
Homo sapiens	*RPL13A*	TCGTACGCTGTGAAGGCATC	TTTTGTGGGGCAGCATACCT
Homo sapiens	*CCN2*	OriGene (HP205671)	
Canis lupus familiaris	*Gapdh*	TTCCACGGCACAGTCAAG	ACTCAGCACCAGCATCAC
Canis lupus familiaris	*Ccn2*	TTTAGGAACAGTGGGAGAGC	CATGAAGAAGGCTGGAGAAC
Canis lupus familiaris	*Gcgr*	TGCTCTTTGTCGTCCCCTGGG	GTAGGATCCACCAGAACCCC

SUPPLEMENTARY FIGURE LEGENDS

Supplementary Figure 1. Flow cytometry gating strategy for immune cell populations in the kidneys of age-matched control mice (control), diabetic high fat-diet fed mice (DMHFD), or DMHFD mice treated with finerenone (100mg/kg diet) in high fat diet for the final 2 weeks (DMHFD + Finerenone).

Supplementary Figure 2. RNAscope in situ hybridization for *Glp1r*, *Gipr* and *Gcgr* in kidney sections from age-matched control mice (control), diabetic high fat-diet fed mice (DMHFD), or DMHFD mice treated with finerenone (100mg/kg diet) in high fat diet for the final 2 weeks (DMHFD + Finerenone). Representative of $n \geq 4$/group. Original magnification x400. Scale bar = 50μm.

Supplementary Figure 3. RNAscope in situ hybridization for *Glp1r*, *Gipr* and *Gcgr* in cardiac sections from age-matched control mice (control), diabetic high fat-diet fed mice (DMHFD), or DMHFD mice treated with finerenone (100mg/kg diet) in high fat diet for the final 2 weeks (DMHFD + Finerenone). Representative of $n \geq 4$/group. Original magnification x400. Scale bar = 50μm.

Supplementary Figure 4. qRT-PCR for *Glp1r* and *Gcgr* in vascular smooth muscle cells (VSMCs; A) and MDCK cells (B) respectively, treated with 100nmol/L aldosterone in the presence or absence of 5μmol/L finerenone (n=6/condition). Values are mean ± S.D.. **P*<0.05, ***P*<0.01 by one-way ANOVA followed by Fisher's least significant difference test.

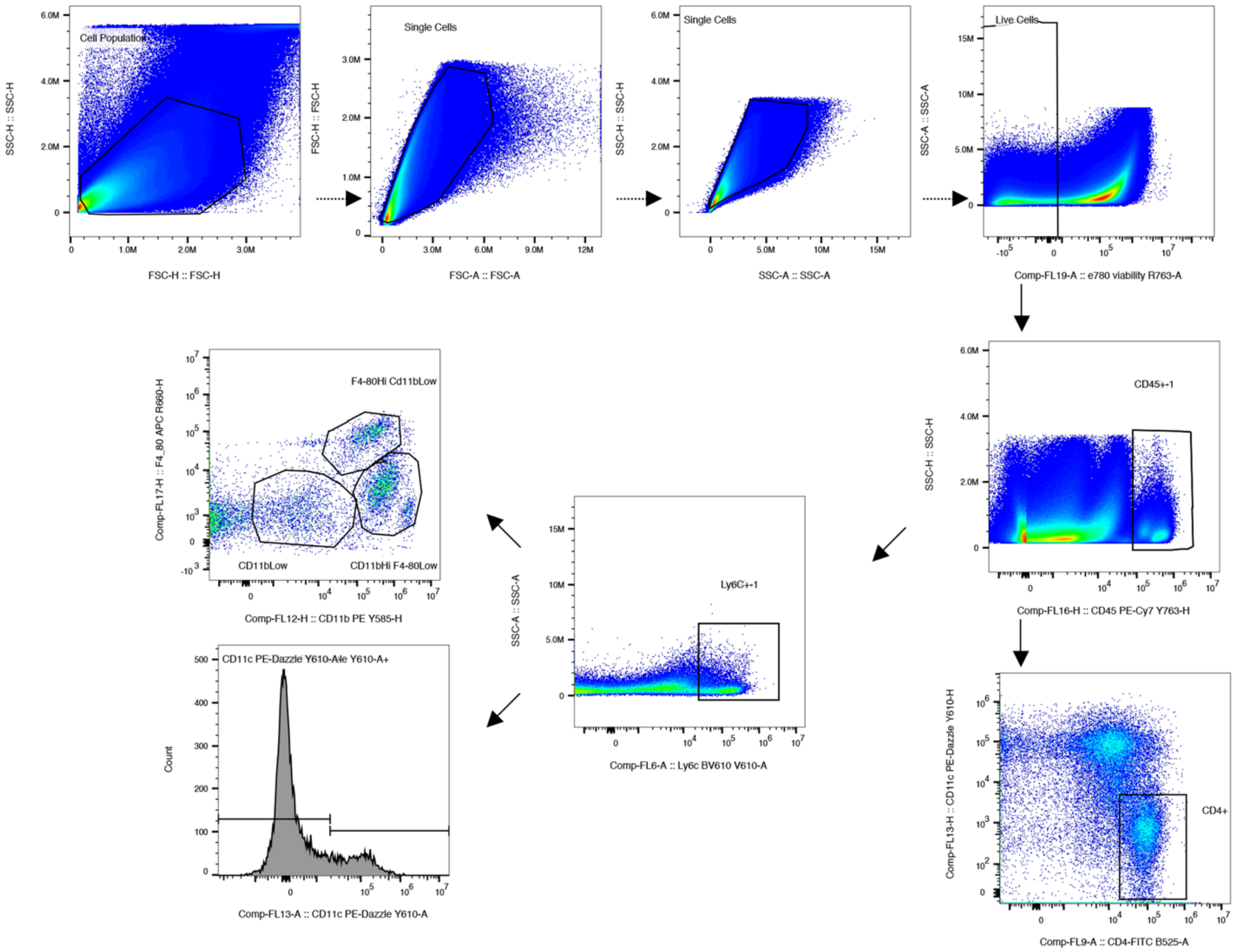

Supplementary Figure 1.

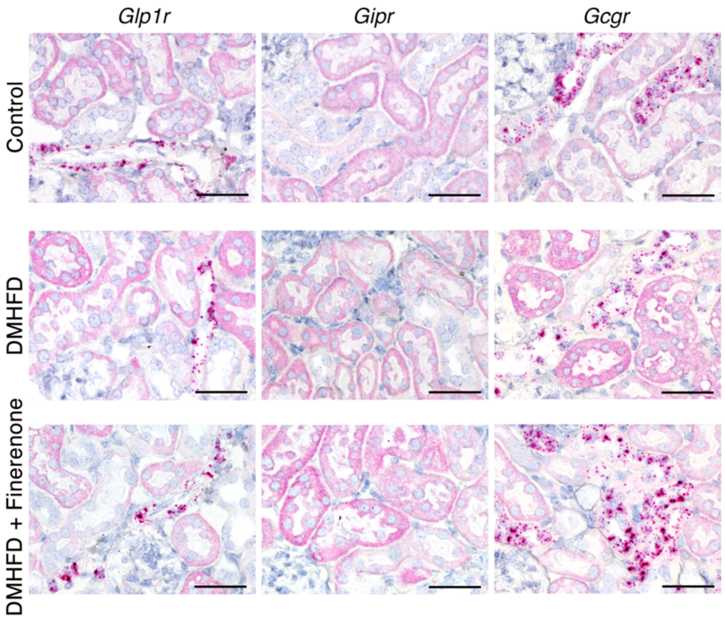

Supplementary Figure 2.

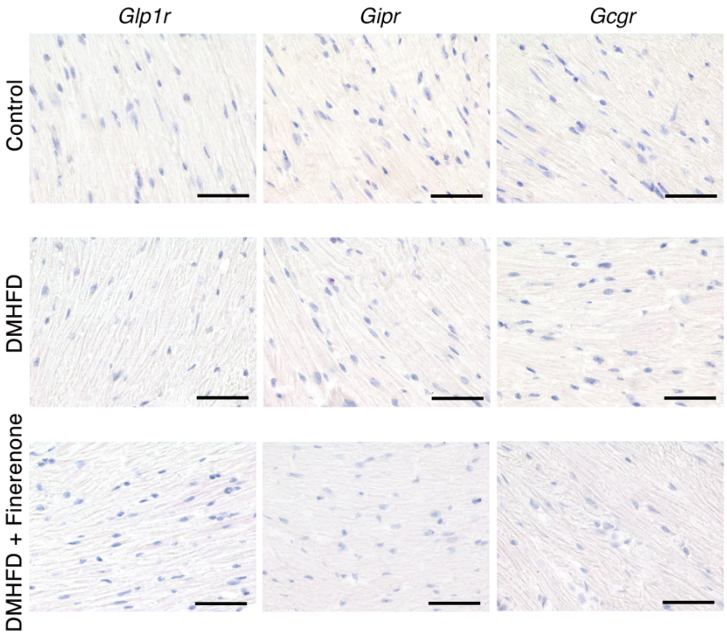

Supplementary Figure 3.

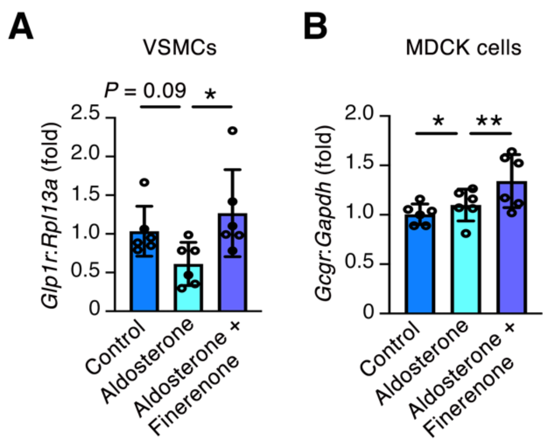

Supplementary Figure 4.
